# Pituitary Apoplexy Precipitated by Systemic Chemotherapy

**DOI:** 10.7759/cureus.23004

**Published:** 2022-03-09

**Authors:** Forrest A Hamrick, Matthew C Findlay, Robert C Rennert, Karol P Budohoski, William T Couldwell

**Affiliations:** 1 Neurosurgery, University of Utah, Salt Lake City, USA

**Keywords:** macroadenoma, chemotherapy, pituitary adenoma, apoplexy, pituitary

## Abstract

Pituitary apoplexy often occurs in patients with previously undiagnosed pituitary adenomas and no predisposing factors. Among patients with precipitating events, there are very few cases of pituitary apoplexy occurring in the setting of systemic chemotherapy treatment. A 31-year-old man with newly diagnosed metastatic testicular cancer developed headaches, nausea, and a right-sided visual field deficit one week after initiation of bleomycin, etoposide, and cisplatin chemotherapy. Computed tomography and magnetic resonance imaging revealed hemorrhage within a pituitary macroadenoma consistent with pituitary apoplexy, and he underwent urgent transnasal resection. We also review the four prior cases of pituitary apoplexy temporally associated with the administration of systemic chemotherapy.

## Introduction

Pituitary apoplexy (PA) is a rare but emergent medical condition in which a patient experiences infarction or hemorrhage within the pituitary gland. Common clinical manifestations of PA include endocrine dysfunction, nausea, headache, ophthalmoplegia, and visual disturbances, with the condition confirmed through magnetic resonance imaging (MRI) or computed tomography (CT) imaging [1]. Although the pathophysiology of PA is poorly understood, it generally occurs in the setting of a pituitary adenoma, wherein tumor growth may outpace vascular supply and cause a hemorrhage or hemorrhagic infarction [1]. However, PA can also occur in a non-adenomatous pituitary gland [1]. PA can occasionally be linked to a precipitating factor, such as major surgery, radiotherapy, coagulopathies, hypertension, pregnancy/postpartum state, and head trauma, and medications such as anticoagulants, estrogen therapies, dopamine agonists, and gonadotropin-releasing hormone (GnRH) agonists [1-3]. However, most cases of PA appear to be spontaneous and present without an obvious precipitating factor.

To the best of our knowledge, there have been only four prior case reports of PA temporally associated with the administration of systemic chemotherapy [4-7]. We add to this literature by reporting the case of a patient who experienced PA one week after initiation of chemotherapy for metastatic testicular cancer.

## Case presentation

**Figure 1:**
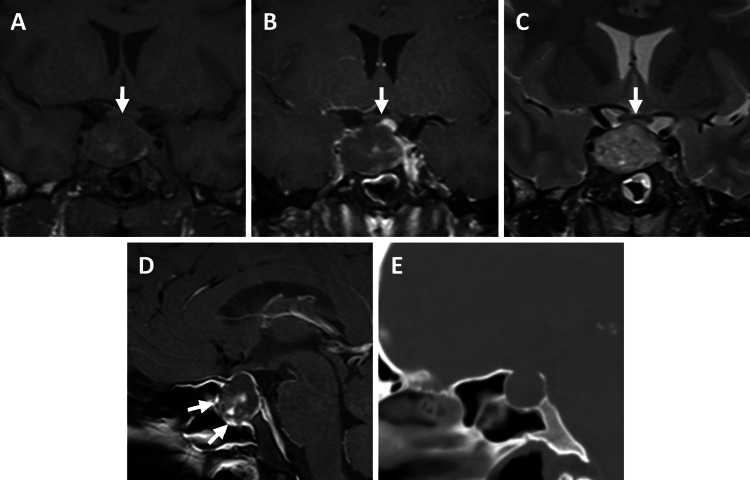
MRI and CT of the head demonstrated a 2.0×2.8×2.1-cm heterogeneously enhancing sellar and suprasellar mass Coronal T1-weighted noncontrast (A); T1-weighted postcontrast (B); and T2-weighted (C) MRI show upward displacement of the optic chiasm (white arrows) and internal blood products. The pituitary stalk and gland are displaced to the left, best seen on postcontrast T1-weighted imaging. Sagittal post-contrast T1 MRI (D) and CT (E) demonstrated expansion of the sella consistent with a prior slow-growing tumor. Enhancement of the sphenoid sinus mucosa is also seen (D; gray arrows), which has been linked to higher intrasellar pressures and more severe PA [8].

A 31-year-old man with a recent diagnosis of seminomatous germ cell testicular cancer with metastases to the pelvis and retroperitoneum (elevated serum lactate dehydrogenase and beta-human chorionic gonadotropin on diagnosis; normal alpha-fetoprotein) presented to the emergency department with several days of severe, progressive bifrontal headaches, nausea, and right-sided blurry vision one week after initiating bleomycin, etoposide, and cisplatin chemotherapy. On examination, he was neurologically intact except for decreased right temporal visual acuity. Head CT and MRI demonstrated a 2.0×2.8×2.1-cm, expansile, heterogeneously enhancing sellar and suprasellar mass displacing the optic chiasm with evidence of internal hemorrhage concerning for apoplexy (Figure 1). Laboratory analysis revealed hyponatremia (Na 132) but no other significant electrolyte, hematologic, or coagulation abnormalities. Pituitary hormone samples were drawn, and he was given fluids and stress dose steroids before he underwent an urgent transnasal transsphenoidal surgery. Partially necrotic and hemorrhagic tissue under pressure was encountered upon dural opening and resected completely with preservation of the pituitary stalk and gland. An abdominal fat and fascia graft was used for closure. The pathological analysis confirmed a partially necrotic and hemorrhagic adenoma with rare growth hormone (GH)- and adrenocorticotropic hormone (ACTH)-positive tumor cells, with a low proliferation rate (MIB1 <2%).

Postoperatively, the patient’s headache resolved and his blurry vision subjectively improved. His pre and postoperative Day 2 pituitary hormone values were notable for secondary adrenal insufficiency (8 AM cortisol 0.8 ug/dL, ACTH 4.9 pg/mL), elevated insulin-like growth factor-1 (554 ng/mL preoperatively and 423 ng/mL postoperatively) with a normal GH level, and low thyroid-stimulating hormone (0.33 mU/L) with a normal free T4. He was discharged home on postoperative Day 4, neurologically stable on maintenance hydrocortisone.

The patient resumed his chemotherapy two weeks postoperatively and underwent a radical orchiectomy two months later. At one-month neurosurgical follow-up, his visual fields were full, and he remained otherwise neurologically intact. Endocrinologic follow-up is ongoing.

## Discussion

We discuss a case of PA in a young patient with a previously undiagnosed pituitary adenoma that occurred after the initiation of systemic chemotherapy in relation to prior literature.

Pituitary adenomas are relatively common, comprising approximately 15% of all intracranial tumors and occurring in more than 20% of the population [9-11]. Although PA may occur in up to 26% of pituitary adenomas, many of these events are subclinical [1,12-13]. Acute, symptomatic PA is rarer and does not have a clear age, sex, or adenoma histologic subtype association [12]. The incidence of PA has nonetheless been found to peak in the fifth and sixth decades of life and is often the first manifestation of an underlying tumor [1,12]. Although larger tumor size is thought to increase risk, both macro and microadenomas can cause PA [1]. Hemorrhage within the sella/suprasellar space can compress the pituitary gland/stalk and cause hypopituitarism and similarly compress the optic nerves superiorly or cavernous sinus laterally and cause visual disturbances. Accordingly, the presence of sphenoid sinus mucosal enhancement on imaging, as seen in this case (Figure 1) and a proxy for higher intrasellar pressures, has been linked to more severe PA and worse neurological and endocrinologic outcomes [8]. Headaches are thought to occur from meningeal irritation [1]. Management of PA initially is focused on correcting fluid/electrolyte imbalances and replacing corticosteroids to ensure hemodynamic stability, with urgent surgery indicated for patients with visual compromise, and/or clinical or neurologic instability [1].

Because only a minority of PA cases have an identifiable predisposing factor, the exact pathophysiology of PA remains unclear [12,14]. Among the previously listed risk factors for PA [1-3], the potential pathophysiologic cause can be categorized as primarily ischemic (surgery, radiotherapy), hemorrhagic (coagulopathies/anticoagulation, hypertension, trauma), or endocrinologic (pregnancy, estrogen therapy, dopamine/GnRH agonist), with the potential for mixed etiologies such as ischemia/hemorrhage possibly with major blood loss from surgery causing both hypotension and coagulopathies. Within the endocrinologic category, possible mechanisms include increased metabolic activity of tumor cells (GnRH agonists) and increased pituitary volume/blood flow (pregnancy/estrogen therapies) [4].

To our knowledge, there are only four other cases of PA associated with the administration of systemic chemotherapies [4-7] (Table 1). Consistent with the broad epidemiology of PA in general, these prior reports include two male and two female patients who ranged in age from 41 to 70 years. The cancers being treated included two hematologic malignancies (chronic myelocytic leukemia, acute myeloid leukemia) and two solid tumors (metastatic breast cancer, metastatic penile squamous cell carcinoma). The chemotherapy regimen in these cases was variably reported, but the timing of the PA development ranged from the first to the third cycle. Two of the four patients underwent surgery for PA (because of visual acuity changes), with postoperative improvement in symptoms reported in both patients. Endocrinologic presentations and outcomes were under-reported in these prior cases. The current case of a 31-year-old man underscores the wide age range potentially affected by PA, as well as the good neurologic outcomes possible with prompt treatment for visual compromise. Including our case, three of the five cases of chemotherapy-associated PA occurred during the first cycle of treatment, suggesting the highest risk of PA may be during the initial treatment round.

**Table 1:** Summary of reported cases of chemotherapy-associated pituitary apoplexy AC: doxorubicin/cyclophosphamide; ACTH; adrenocorticotropic hormone; BEP: bleomycin, etoposide, and cisplatin; CMV: cisplatin, methotrexate, and vinblastine; CN: cranial nerve; CTX: chemotherapy; F: female; GH: growth hormone; HA: headache; hemorr.: intratumoral hemorrhage; HT: hypertension; M: male; N: No; NF: nonfunctioning; NR: not reported; OD: right eye; OS: left eye; OU: both eyes; PA: pituitary apoplexy; PHP: panhypopituitarism; PRL: prolactinoma; s/p: status post; TTP: thrombocytopenia; Y: yes

Author	Age/ sex	Known adenoma	Oncologic diagnosis	CTX regimen; timing of PA	Generalized symptoms	Visual symptoms	Endocrine dysfunction	Hematologic/ coagulation abnormalities	Adenoma size/ imaging findings	Adenoma function	Surgery	Visual outcome	Endocrinologic outcome
Davies et al. 1998 [7]	70M	N	Metastatic penile squamous cell carcinoma	CMV; during 2nd cycle	HA, nausea, vomiting	Partial left CN III palsy	Partial anterior hypopituitarism	None	Macro with hemorr.	NF	No	Resolution of CN III palsy	Discharged on hydrocortisone
Silberstein et al. 2008 [6]	55M	N	Acute myeloid leukemia	NR; day 6 after induction therapy	HA, photophobia, nausea, fever	None	PHP	TTP, mild coagulopathy	Macro with hemorr.	NF	No	NR	PHP
Jang et al. 2018 [5]	41F	N	Metastatic breast cancer	AC; beginning of 3rd cycle	HA, vomiting	Left CN VI palsy, hand motion OS, temporal hemianopsia OD	None	None	2.8-cm solid and cystic macro with hemorr.	NF	Yes	Near-complete resolution of all visual symptoms	NR
Maki et al. 2018 [4]	64F	Y: PRL s/p prior surgery, on cabergoline	Chronic myelocytic leukemia	NR; during 1^st^ cycle	HA, loss of appetite, fever, HT	Partial right CN III palsy, left CN VI palsy, blurry vision, scattered OU visual field defects	NR	TTP	Macro with hemorr.	PRL	Yes: 5 days after presentation, after correction of TTP	Resolution of all visual symptoms	NR
This paper	31M	N	Metastatic testicular cancer	BEP; 1 wk after initiation of 1^st^ cycle	HA, nausea	Decreased OD visual acuity	Partial anterior hypopituitarism	None	2.8-cm macro with hemorr.	Rare GH and ACTH + cells	Yes	Resolution of visual defect	Discharged on hydrocortisone

Given the limited prior reports, the mechanism behind chemotherapy-associated PA has not been explored. Some chemotherapy regimens are known to cause hematologic and coagulopathic abnormalities, which can independently increase the risk of PA. This was seen in two of the four previously reported patients [4,6], both with hematologic malignancies that can also impact bleeding risk but not in the present case. Moreover, although there is less data addressing the effects of chemotherapy on pituitary function, most evidence suggests it plays an indirect or minor role in altering the hormonal axis [15-16]. Although adenomas are benign tumors with generally low proliferation rates, potential mechanisms of PA after chemotherapy may be related to cytotoxic effects on the tumor and/or disruption of its blood supply, causing infarction.

Given the complex array of side effects that often accompany chemotherapy treatment and the potential for PA to present insidiously rather than acutely [17-18], the incidence of PA in patients in this setting is likely underappreciated. In the absence of formal guidelines, because the risk of chemotherapy-related PA is theoretically increased with macro- versus microadenomas, we agree with previous authors that prophylactic surgical removal may be considered for patients with known or incidentally identified larger pituitary tumors requiring cytotoxic chemotherapies to reduce PA risk [5]. This decision must nonetheless balance the oncologic risk of delaying chemotherapy. Close monitoring of patients with pituitary adenomas of any size is also prudent during chemotherapy treatment.

## Conclusions

Systemic chemotherapy can rarely precipitate PA in patients with pituitary adenomas, with a total of five reported cases in the literature. Mechanistically, this is likely related to chemotherapy-induced coagulopathies and/or the cytotoxic or ischemic effects of chemotherapy on the tumor. Treatment of chemotherapy-related PA follows standard treatment algorithms, with initial medical stabilization and surgery for patients with visual/neurologic defects. In patients with larger macroadenomas, prophylactic resection may be reasonable before the initiation of systemic chemotherapies.
